# Supplementary material for: Investigation and Analysis of Wettability, Anisotropy, and Adhesion in Bionic Upper and Lower Surfaces Inspired by Indocalamus Leaves
Source: Molecules. 2024 Jul 23;29(15):3449. doi: 10.3390/molecules29153449 (PMC11313824; doi:10.3390/molecules29153449)
Supplement: Supplementary file 1 [file molecules-29-03449-s001.zip › molecules-3085866-supplementary.pdf]

Supplementary material

Investigation and Analysis of Wettability, Anisotropy, and Adhesion in Bionic Upper and Lower Surfaces Inspired by Indocalamus Leaves

Bo Wang [1,2], Donghui Chen [1,2,*], Xiao Yang [1,2] and Ming Li [3]

[1] Key Laboratory of Bionic Engineering, Ministry of Education, Jilin University, Changchun 130022, China; wanborn@163.com (B.W.); yangxiao22@jlu.edu.cn (X.Y.)
[2] College of Biological and Agricultural Engineering, Jilin University, Changchun 130022, China
[3] School of Mechanical and Aerospace Engineering, Jilin University, Changchun 130022, China; lmingdr@163.com
* Correspondence: dhchen@jlu.edu.cn

Table S1. Surface roughness of upper and lower surfaces of the indocalamus leaf.

Surface	Sa (μm)	Sz (μm)	Sq (μm)	Sdr (%)
Upper surface	6.865	67.216	8.586	67.229
Lower surface	9.570	148.003	12.186	93.801

Figure S1 displayed the schematic diagram and the testing example of the dynamic adhesion test. A 5 μL water droplet was suspended at the syringe tip, and the platform with the testing sample was slowly rising at a speed of 0.5 mm/min until contacted and compressed the water droplet. Then, the platform was moved down until the water droplet and surface were completely separated. The entire testing process was divided into five steps: initial state, begin contact, severe contact, last contact and final state. The adhesion force can be calculated by the analysis software.

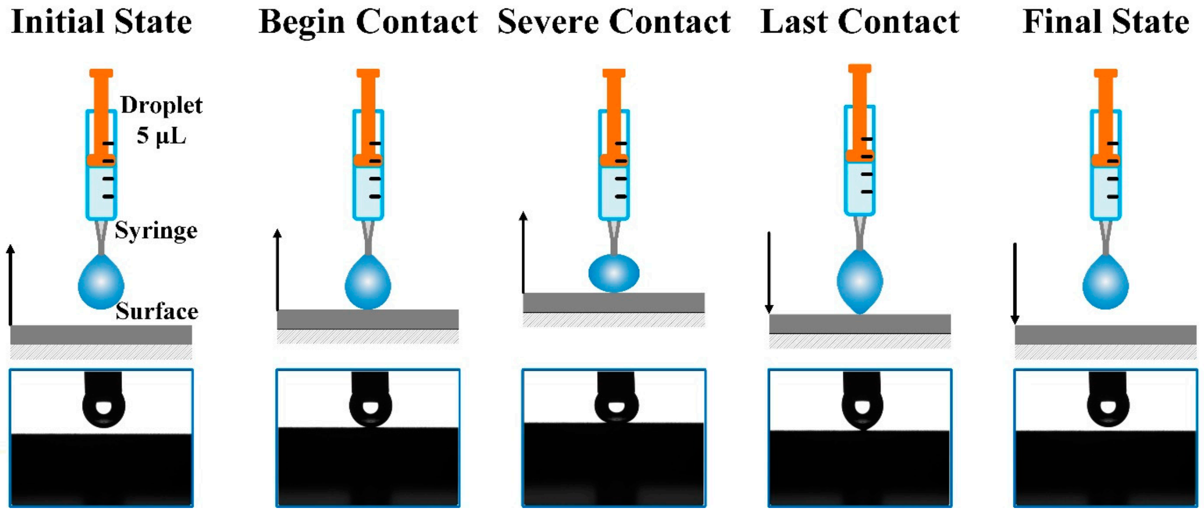

Figure S1. Schematic diagram and the testing example of the dynamic adhesion test.

As shown in Figure S2, the SEM images and CLSM images of BUS_{200} and BLS_{200} modified by FAS-17 are similar to those before modification (Figures 4b and 5b in the manuscript). And the roughness of both the surfaces before and after modification has hardly changed (Table S2). Consequently, changes in the chemical composition of the BUSs and BLSs play a crucial role in the wettability, anisotropy and adhesion of bionic surfaces.

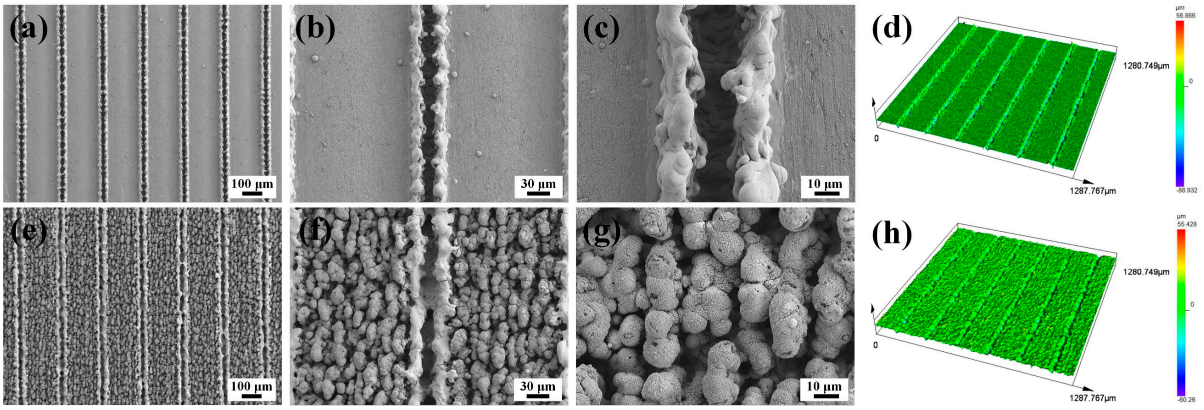

Figure S2. Morphologies of the BUS$_{200}$ and BLS$_{200}$ modified by FAS-17. (a-c) SEM images of BUS$_{200}$; (d) CLSM images of BUS$_{200}$; (e-f) SEM images of BLS$_{200}$; (h) CLSM images of BLS$_{200}$.

Table S2. Surface roughness of BUS$_{200}$ and BLS$_{200}$ before and after modification.

Surface	Sa (μm)	Sz (μm)	Sq (μm)	Sdr (%)
BUS$_{200}$	2.383	105.888	4.394	62.631
Modified BUS$_{200}$	2.183	105.394	4.157	56.899
BLS$_{200}$	5.785	152.086	7.861	85.432
Modified BLS$_{200}$	5.859	151.435	7.953	85.695

To evaluate the surface stability, we subjected the BUS$_{200}$ and BLS$_{200}$ to abrasion test with sandpaper. Figure S3a displayed the schematic diagram of the abrasion test. The 1000# sandpaper was used as the friction plate, and the weight of 200g was used as the normal load and the abrasion distance was 10 cm. Fig. S3b illustrated the changes of CA$_{\perp}$s and CA$_{//}$s on the BUS$_{200}$ and BLS$_{200}$. After 20 times of abrasion, the CA$_{\perp}$ and CA$_{//}$ on the BUS$_{200}$ decreased to 153.0±2.0° and 150.9±0.6°, and the CA$_{\perp}$ and CA$_{//}$ on the BUS$_{200}$ decreased to 154.7±0.8° and 153.1±0.2°. The results showed both the surfaces still possessed superhydrophobicity. Consequently, both the BUSs and BLSs possessed excellent mechanical stability, which can be widely used in many extreme work environments.

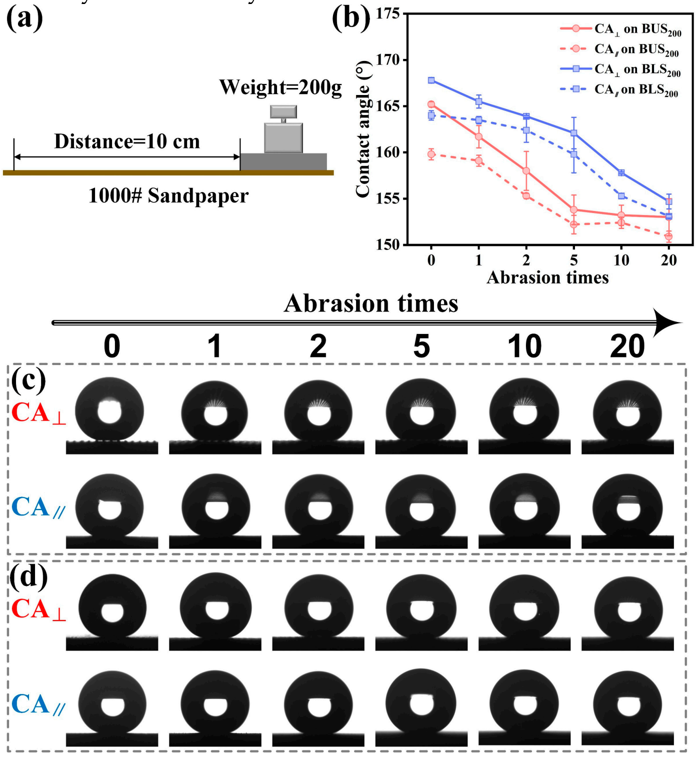

Figure S3. Abrasion test of the BUS_{200} and BLS_{200}. (a) Schematic diagram of the abrasion test; (b) Changes of the $CA_{\perp}$s and $CA_{//}$s of the BUS_{200} and BLS_{200} with the increase of abrasion times; (c) and (d) Contact angle image corresponding to the values in Figure S3b.

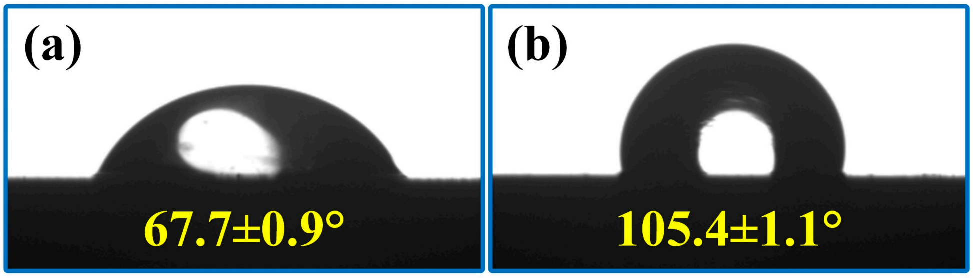

Figure S4. CAs of the water droplet on the polished surface. (a) Without chemical modification; (b) After chemical modification.

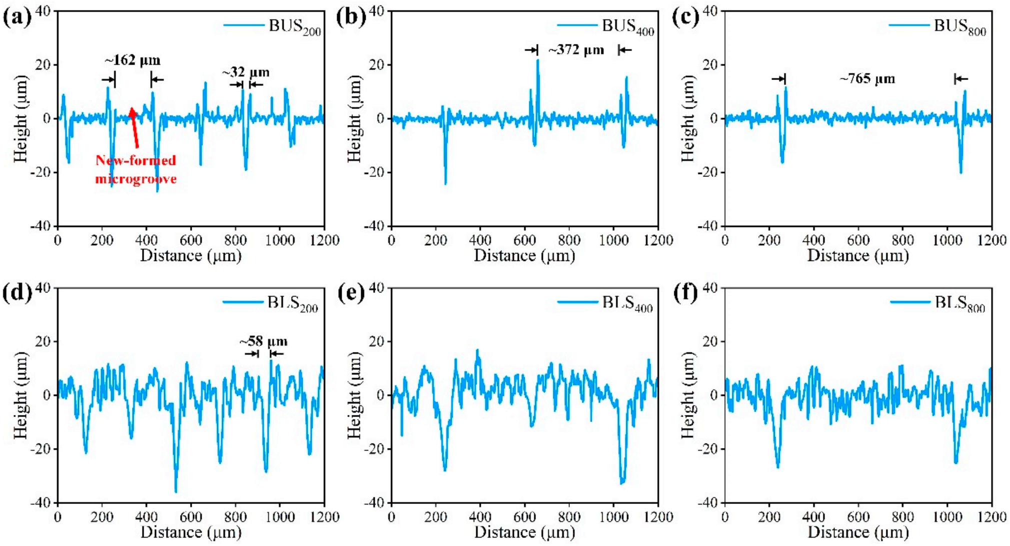

Figure S5. Height of the cross-section profile lines of BUSs and BLSs. (a-c) BUS_{200}, BUS_{400} and BUS_{800}; (d-f) BLS_{200}, BLS_{400} and BLS_{800}.

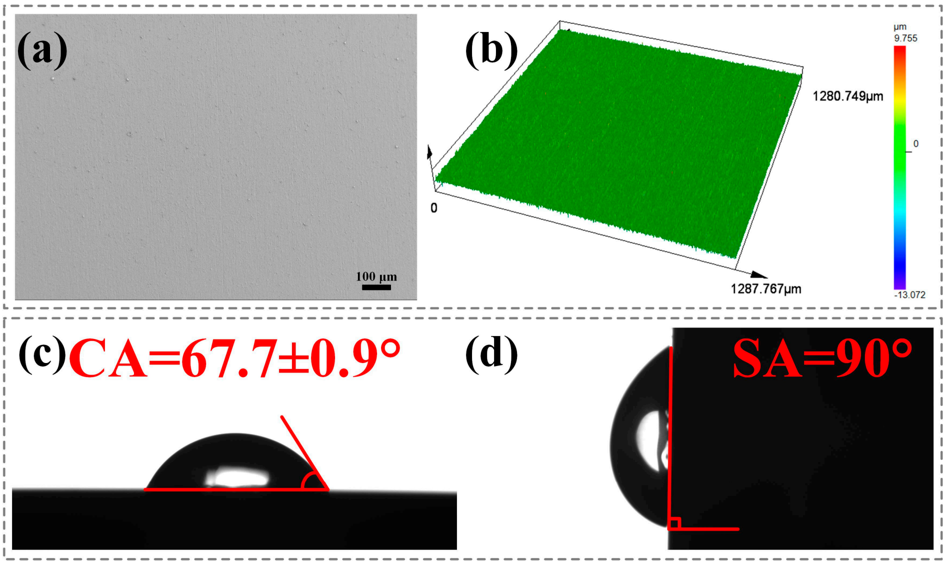

Figure S6. Morphologies and wettability of polished surface. (a) and (b) SEM and CLSM images of the polished surface; (c) and (d) CA and SA of the polished surface.
